# Editorial Notes

**Published:** 1886-07

**Authors:** 


### Editorial Notes.

*To Our Subscribers.*—This number begins Vol. II. All who began with the July number last year are notified *now* of the expiration of their subscription, and it is *earnestly hoped* they will *promptly* renew, without waiting to have bills and duns sent them. We ask, need, and cordially and earnestly invite your *material* as well as moral support. *You know* a Journal can not be conducted without money. What more need we say to those who *intend* to aid us, and who want us to continue the Journal? Send along the money.

*Dr. J. B. Hamilton, Surgeon-General, M. H. S.*, has accepted the professorship of Surgery in the Chicago Polyclinic. He will not resign his government position, but will spend his vacation in Chicago.

*The Late Dr. Burt.*—We are much pleased to state that Dr. Burt had a paid-up policy in the Equitable Life Assurance Society for $3,585, and also held a policy in the Knights of Honor for $2,000, and one in the Knights and Ladies of Honor for $3,000. Dr. Burt was not a member of the Physicians' Mutual Benevolent Association of Texas.

*Notice to Advertisers—A Rare Chance.*—The fourth cover page, now occupied by the Peacock Chemical Company, can be secured for a year; possession given with next issue—August. As that is the only cover or preferred space not taken, you would do well to apply at once.

*Dr. A. G. Clopton*, on June 16, delivered before the faculty of the Texas University, the Alumni Association, and a large audience of ladies and gentlemen, an address on the Life and Character of Dr. Ashbel Smith, which was a master piece of English composition, and delivered in the Doctor's well known forcible and graceful manner. He paid a handsome, glowing tribute to the memory of this good man. The address reflects great credit upon the distinguished orator, and we would be pleased to reproduce it entire, if we had the room. It is published in full in the *Iron News*, of Jefferson, Texas.

---

*Accident to Dr. Herff.*—We are pained to learn that a serious accident recently befel the distinguished Western surgeon, Dr. Ferdinand Herff, of San Antonio. He has a summer residence at Boerne, to which he repairs in the hot months to seek much-needed rest from his laborious practice,—and during his sojourn at this retreat, we learn, he refuses to see patients (very properly). A short while ago he was thrown from his buggy, and it is said he received such serious injuries that it is feared he will not recover.

---

*The Remedy worse than the Disease.*—One Dr. Villavicencio proposes to practice inoculation for yellow fever in New Orleans, and the board of health of that city proposes to fumigate him if he attempts it.—*N. Y. Med. Times.*

The board is afraid that some of the pent up *cryptococci* might escape, and finding a nidus in the city atmosphere, multiply and set up an epidemic. *Quis custodiet ipsos custodes?*

---

*Death of Dr. Joe Willis.*—Dr. Joseph S. Willis, of Waco, Texas, died from the effects of an overdose of bromidia, taken for the relief of pain (so stated in the telegram to the *Statesman*), on the evening of July 6th. He was 38 years old, and had been for many years a member of the Texas State Medical Association, and was a leading practitioner in central Texas.

---

The title page for Vol. One is sent out with this issue. Send it to the binder with your Journals.

---

*Amenities of Medical Journalism "Down East."*—Brothers Shrady, of the *New York Medical Record*, and Brinton, of the Philadelphia *Medical and Surgical Reporter*, have locked horns, so to speak, and are 'rastlin' over an accusation which the former makes, that Dr. Brinton, while defending the Code, is virtually violating it in furnishing a certificate of exceellence of a "certain water." Brinton denies the charge, and says he can prove that the proprietors of said water are using his name without authority; and applies the following choice epithets to his "esteemed contemporary" of the *Record:* "Disreputable and indecent." * * "And we apply these adjectives to Dr. Shrady's action in the case quoted."

Shrady is yet to be heard from. Such language between editors of leading medical journals "down East, may be considere "reputable and decent," and may pass, perhaps, unnoticed; but down this way it would indicate the probability of early obsequies.

---

*Another Veteran Texas Physician Gone.*—We are pained to chronicle the death of Dr. David Sayres, of Bastrop, who died at his home, in that place, Friday, July 2, aged 75 years.

Dr. Sayres was born in Wythe county, Virginia, in 1811; graduated at the University of Pennsylvania in 1835; settled in Mississippi the next year, at Grenada; was there married in 1840 (name of lady unknown); removed to Texas in 1851, and, having lost his first wife, was married in 1854 to Miss Inda Scott. He was the father of Congressman Joe Sayres, of this district.

Dr. Sayres was a member of the Physicians' Mutual Benefit Association of Texas, and his death is the second that has taken place in that order since its organization in 1884. He was a splendid type of the old Virginia gentleman, and a true physician. His death is much regretted by a host of warm friends and by the entire community where he resided.

---

*Yellow Fever Commission..*—Dr. Holt's bill, as passed the Senate, is so modified as to leave out the appointment of any one from civil life as a member of the commission. The National Board got in their work partly, at least, and it looks like a repetition of the "dog in the manger story—if I can't have ti, you shall not." The bill provides for a commission of two officers already in the government service, with authority to employ expert service, nothing more; so we learn from the *Sanitary News* of July 10.

———

*The First Inoculation in America.*—The first inoculation undertaken in America to prevent the occurrence of hydrophobia, according to the plan devised by Pasteur, was undertaken last Monday at the Carnegie laboratory, on East Twenty-sixth street, New York city, by Dr. Valentine Mott. The patient was a seven-year-old boy, named Harold Newell, a son of Dr. J. C. Newell, of Jersey City. He was bitten by a pet dog on June 24. Dr. Mott has been making preparations for several weeks past, since his return from Paris, where he was instructed by Pasteur himself, and from whose laboratory virus was brought to New York.

The first application of Pasteur's theory in America is a matter of great imortance and interest, and it is hoped may be successful. It is believed by Dr. Mott and Dr. Newell that the dog which bit Harold was suffering from rabies at the time. Inoculations will be made daily until fourteen are performed, when the operation will have been completed.—*Sanitary News*, *July* 10.

We learn that the patient had such alarming symptoms that the inoculation was abandoned.

———

*A Good Move.* We are much gratified to learn through the *N. O. Medical and Surgical Journal*, of the organization of "The Physicians' Mutual Benevolent Association, of Louisiana" Dr. Richard H. Day, ex-President of the Louisiana State Medical Association, one of the oldest and most respectable physicians of the State, and Dr. J. W. Dupre, ditto, ditto, are at the head of it; the former being President and the latter Secretary. A charter has been obtained and a call issued to the profession of Louisiana, to rally to the support of the truly benevolent scheme. We observe the death benefit assessment has been placed at $3 instead of $1, as it is in the Texas P. M. B. A; and the initiation fee or yearly dues $1, the same as in the latter. We wish our noble brethren Day and Dupre every success in this their good work. They are both members of the Texas organization.

**It is a shame that the movement in Texas has not received a more cordial support.**

